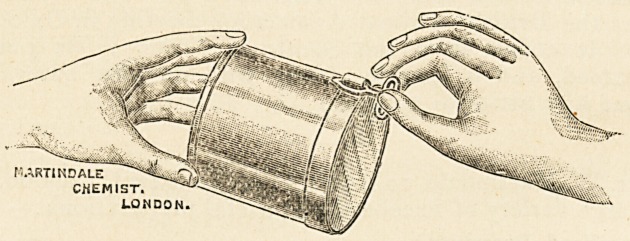# Notes on Preparations for the Sick

**Published:** 1905-03

**Authors:** 


### Notes on Preparations for the Sick.

**Tabloids: Reduced Iron Compound; Reduced Iron and Rhubarb Compound.**—Burroughs, Wellcome & Co., London.—A convenient means of administering reduced iron with extract of nux vomica is afforded by these tabloids. Each contains:—

| | |
|---|---|
| Reduced Iron ... ... ... ... ... | gr. 2 |
| Extract of Hyoscyamus ... ... | gr. 1 |
| Extract of Nux Vomica ... ... | gr. ½ |
| Oil of Caraway ... ... ... ... | min. ¼ |

In practice it is often convenient to prescribe a slight variation of this formula in order to combat indigestion and constipation. For this purpose "Tabloid" Reduced Iron and Rhubarb Compound is issued. Each contains:—

| | |
|---|---|
| Reduced Iron ... ... ... ... ... ... | gr. 2 |
| Extract of Hyoscyamus ... ... | gr. 1 |
| Extract of Nux Vomica ... ... | gr. ½ |
| Compound Rhubarb Pill ... ... | gr. 1 |
| Oil of Caraway ... ... ... ... | min. ¼ |

It will be observed that both products contain the same doses of reduced iron and extract of nux vomica, and both may be therefore ordered for a patient with a direction to take one or other, or both, in such proportions as from time to time meet the digestive difficulties of the case.

---

**Effervescent Laxative Lithia; Elixir Pepsin, Bismuth and Iron.**—Parke, Davis & Co., London.—The effervescing compound is another of the numerous combinations of lithia with alkaline salts which are so popular at present as anti-gouty laxatives. Each spoonful dose contains five grains of lithia citrate with citrate and phosphate of potassium and sodium.

The elixir is a very useful and agreeable combination of saccharated pepsin (U.S.P.) with bismuth and ammonium citrate and soluble ferric pyrophosphate.

---

**Glyco-Camph. Co. Buxton.**—T. BUXTON & Co., Queen's Road, Clifton.—This is a modification of the ancient and popular preparation commonly called "The Infirmary Cough Syrup." It is equivalent to the syr. camph. co. (B.R.I.), but with glycerine in place of syrup, a very useful modification for the many cases in which there are positive objections to sugar.

**Aseptic Nasal Douche.**—This is essentially the same as the K and O glass douche which has so long been in use in the United States. It is very simple, and it renders easy the formerly troublesome operation of washing out the nasal chamber.

---

**Antiurique de Pontac; Colchique de Pontac; Baume de Pontac.**—W. EDWARDS & SON, 157 Queen Victoria Street, London.—These soulagements de Pontac have been devised for the treatment of gout. The antiurique is an effervescing combination of piperazine with alkaline benzoates. The colchicum preparation is a standardised form of colchicine prepared from the fresh flowers of Vosgian colchicum. The Baume is a pommade for external use, composed of synthetic methyl salicylate with some of the opium salicylates; it is mounted in collapsible tubes very convenient for use. These Pontac remedies have given good results in the hospitals of Paris, but are not yet much known in this country.

---

**Diastase - Setterie.**—THE BRITISH PHARMACAL COMPANY, London.—This malt extract contains the active digestive ferment of pure barley malt. Its diastasic activity is said to be greatly in excess of all the usual commercial extracts of malt, in the proportion of $300^{\circ}$ against $24^{\circ}$. Its chief use is to enable the mother to make at home a malted food to serve as an aid to digestion and to facilitate the assimilation of starchy matter. It is said to be invaluable in making malted foods for infants in amyl-dyspepsia and the malnutrition of wasting diseases. It may either be mixed with milk foods, porridge, &c., or it may be taken as a medicine in teaspoonful doses. Examination in the laboratoɹy confirms the statement that it possesses remarkable diastasic power, converting starch into maltose.

---

**Aspirin Effervescens,** gr. x in ʒj; **Sodii Sulpho-Carbolas Effervescens,** gr. x in ʒj; **Cerii Oxalas Effervescens,** gr. x in ʒj.—BLAKE, SANDFORD & BLAKE, 49 Dover Street, Piccadilly, W.—These new effervescing combinations commend themselves as likely to be of considerable utility. The combination with alkaline citrate increases the efficacy of the aspirin, and it also increases the solubility of the cerium salt. They should have quite a wide range of utility and be much in demand.

---

**Fry-Allen and Hanbury's Malted Cocoa.**—J. S. Fry & Sons, Bristol.—This excellent preparation is a combination of Allen and Hanbury's Extract of Malt with Fry's Pure Cocoa Extract, dried at a gentle heat to avoid injury to the diastase. It easily supplies a nutritious and invigorating beverage for breakfast, luncheon or supper. It may be taken either with water or milk, and it does not need to be boiled. A similar malted chocolate paste is also manufactured, and these foods need only to be known to be appreciated.

---

**Milk Food Chocolate** (Allenburys); **Milk Food Cocoa (Peptonised).** — Allen & Hanburys, Ltd. London. — The chocolate has the following composition, as shown by analysis published by the *Lancet*:—

7.62 per cent. of proteid.
33.40 per cent. of fat.
56.41 per cent of milk and cane sugars.

It is made from the purest chocolate, and contains in addition 25 per cent. of the Allenburys Milk Food. Intended primarily as a concentrated and digestible form of nutriment, it is incidentally a wholesome and delicious sweetmeat. Children can easily be given fat in this very palatable form.

On a busy day when regular meals are impossible, or during fatiguing night work, the Allenburys Milk Food Chocolate will be found handy as an "emergency ration."

The Peptonised Milk Cocoa possesses the following special advantages:—

(1) It contains a suitable proportion of milk, and can be quickly and easily made, the addition of hot water only being necessary.

(2) Being peptonised, it can be taken by invalids and children, and by those who cannot take ordinary cocoa.

---

**Manhu Diabetic Foods.**—The Manhu Food Co., Limited, 88 Charing Cross Road, London, W.C.—We have received samples of many varieties of the Manhu foods, including the diabetic flour, biscuits, flaked wheat, flaked barley, prepared barley, barley-cocoa, and diabetic macaroni.

These foods have been much appreciated by certain patients, who have much preferred them to the ordinary starchless glutin foods for diabetics.

This is what the promoters say of their diabetic foods: "The Manhu treatment of diabetes does not consist of drugging the patient, nor of excluding the use of carbohydrates. The starch granules proper to the cereal may be demonstrated under the microscope as still existing in Manhu Diabetic Flour. Its principle is a treatment of the flour which so modifies the molecular conditions of the carbohydrates that they are more easily broken up into carbon dioxide and water, and thus they are made assimilable and reducible in the systems of diabetics, to whom ordinary carbohydrates are a poison. No drugs are given. The aim is to supply agreeable nutrition in such a form as will feed the system of the diabetic. Experience shows that if the diabetic can be kept alive and up to normal weight by means of suitable foods, a natural recovery at length takes place. The Manhu foods do not act as a poison, like ordinary breadstuffs, on the diabetic system. At the same time they will keep a healthy man in full vigour for an indefinite time, while for invalids generally they maintain the strength most admirably."

Micro-chemical examination enables us to confirm the above statement, inasmuch as it is easy to see the numerous starch granules which are undoubtedly present. The diabetic bread was found to give the characteristic reactions of starch. It must be for the physician to decide whether such foods are desirable for the individual case.

---

**Perrier: French Natural Sparkling Table Water.**—PERRIER, New Bond Street, London.—This water attracted much attention at the British Medical Association meeting in Oxford last year. It comes from the South of France; its purity is guaranteed, and it sparkles freely with its own natural gas, without any artificial æration.

---

Mr. W. Martindale, 10 New Cavendish Street, London, sends us a large variety of excellent and useful preparations:—

**Sterilised Dressings**:—

Absorbent wool.

Sterile Bandages, in hermetically sealed tin.

Dried Moss, compressed into sheets, gauze covered, very absorbent.

**Amyl nitrite capsules.**

**Syrupus iodo-tannicus** (gr. ij of iodine in each drachm).

**Maltolivine**, an emulsion of olive oil with malt extract for habitual constipation.

**Lithium hippurate**, effervescing.

" " vescettes, gr. v.

**Lithion**, a granular combination of lithium citrate, mag. sulph. and sod. sulphate.

**Glycaphorm**, a glycerole of diacetyl-morphine hydrochloride, for the relief of cough.

**Lysoform** mouth wash, tooth paste, and pessaries.

These excellent, and for the most part well-known, preparations are all prepared and mounted with great care; their uses are sufficiently obvious, but further details may be found in Martindale's *Extra Pharmacopœia*.

The **amyl nitrite capsules**, glass encased in cotton wool and silk, are invaluable in the treatment of angina, spasmodic asthma, migraine, neuralgia, *post-partum* hemorrhage, hæmoptysis, as an antidote to chloroform, to ward off epileptic attacks, in threatened fainting and other fits, and as a restorative after gas in dental extractions.

Similar glass capsules are manufactured of adrenalin (an economical and handy way of keeping the solution), chloroform, ether, ethyl bromide, ethyl iodide and chloroform combined, of sodium cacodylate, and of distilled water. These latter are convenient for dissolving hypodermic tablets, particularly where the medical man is called out and has reason to doubt the cleanliness of the water.

**Ether soap also ether soap with mercuric iodide** are useful detergent preparations for operative work.

**Iodum oleatum.**—A non-staining iodine and oleic acid compound containing 10 per cent. of iodine.

**Marrubin.**—A bone marrow preparation as a nutrient for children, and in anæmia.

**Lang's Eye Bottle.**—Useful for the consulting room. Stands are supplied; they are either plain or engraved with the solutions usually employed—cocaine, homatropine, atropine, &c.

**Analytical Outfits** for the medical man, including bacteriological test cases, urine test cases, &c.

---

**Furonculine.**—The Zyma Company for Bacteriological Pharmacy, Montreux, Switzerland. General Agents for Great Britain: Messrs. Thomas Christy & Co., London.

It is claimed that this preparation on the basis of dry beer yeast is the only product of its kind in which the cells of the beer yeast have preserved their vitality.

Dr. Du Bois says that it "acts as a powerful stimulant and antiseptic on the intestinal canal, influencing favourably urticaria, acne, and boils, and that its stimulant and antiseptic properties are exhibited in a degree no less remarkable by its action on external ulcerations of a callous nature."

It is not unpalatable, and should therefore be welcomed by those who suffer from these forms of skin disease.

**Antidiabetic yeast** has long been used in the treatment of glycosuria. It is believed to be of value in the treatment of diabetes mellitus, more especially at the commencement of the disease, for it is at this stage that the sugar which is eliminated is derived chiefly from the carbohydrates. In the later stages its merits depend upon its power of allowing a less restricted dietary not only without damage but with positive benefit to the patient. Furthermore, by the chemical modifications to which its activity gives rise in the digestive tract it acts as a powerful intestinal antiseptic, an effect which is of special value to diabetics, who are, as is well known, very susceptible to the slightest intoxication, and who bear such intoxication remarkably badly.

---

**Elixir Cascaræ Sagradæ** (Pitchford).—W. Pitchford and Son, Bristol.—This is a palatable preparation of cascara sagrada, from which the bitter principle has been removed without impairing the aperient properties of the drug.

One special feature is that the sweetening agent is glycerine, not sugar, which is objectionable in certain cases. The flavour of the elixir is due to tincture of orange.

**Syrup. Glycerophosph. Co.** (Pitchford).—An elegant pharmaceutical product, containing amongst other ingredients glycerophosphates of iron, calcium, and magnesium. The glycerophosphates enable the physician to administer phosphorus in a convenient and readily assimilable form.

## Figures and Tables

**Figure f1:**
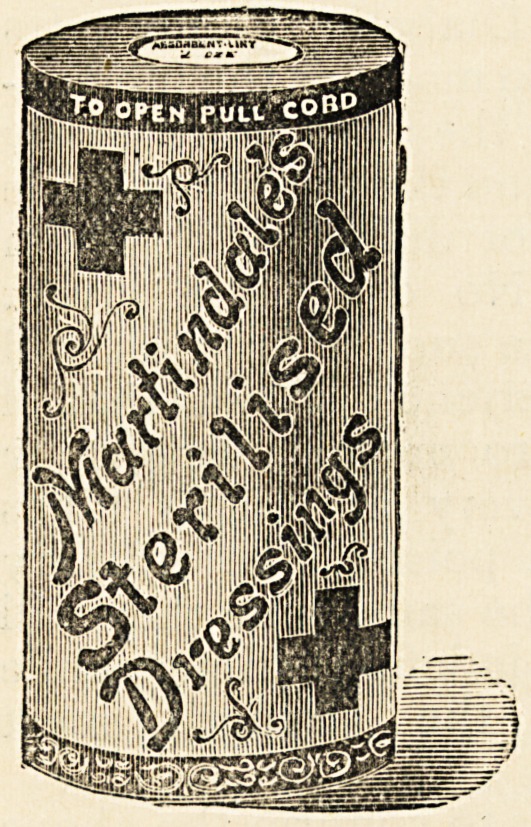


**Figure f2:**